# Tunable Polymeric Mixed Micellar Nanoassemblies of Lutrol F127/Gelucire 44/14 for Oral Delivery of Praziquantel: A Promising Nanovector against *Hymenolepis nana* in Experimentally-Infected Rats

**DOI:** 10.3390/pharmaceutics14102023

**Published:** 2022-09-23

**Authors:** Waleed M. Arafa, Mohammed H. Elkomy, Heba M. Aboud, Mona Ibrahim Ali, Samah S. Abdel Gawad, Shawky M. Aboelhadid, Emad A. Mahdi, Izzeddin Alsalahat, Heba Abdel-Tawab

**Affiliations:** 1 Department of Parasitology, Faculty of Veterinary Medicine, Beni-Suef University, Beni-Suef 62511, Egypt; 2 Department of Pharmaceutics, College of Pharmacy, Jouf University, Sakaka 72341, Saudi Arabia; 3 Department of Pharmaceutics and Industrial Pharmacy, Faculty of Pharmacy, Beni-Suef University, Beni-Suef 62514, Egypt; 4 Department of Medical Parasitology, Faculty of Medicine, Beni-Suef University, Beni-Suef 62511, Egypt; 5 Department of Pathology, Faculty of Veterinary Medicine, Beni-Suef University, Beni-Suef 62511, Egypt; 6 UK Dementia Research Institute Cardiff, School of Medicine, Cardiff University, Cardiff CF24 1TP, UK; 7 Department of Zoology, Faculty of Science, Beni-Suef University, Beni-Suef 62521, Egypt

**Keywords:** praziquantel, *Hymenolepis nana*, polymeric mixed micelles, oral targeting, Box-Behnken design, pharmacokinetics

## Abstract

Hymenolepiasis represents a parasitic infection of common prevalence in pediatrics with intimidating impacts, particularly amongst immunocompromised patients. The present work aimed to snowball the curative outcomes of the current mainstay of hymenolepiasis chemotherapy, praziquantel (PRZ), through assembly of polymeric mixed micelles (PMMs). Such innovative nano-cargo could consolidate PRZ hydrosolubility, extend its circulation time and eventually upraise its bioavailability, thus accomplishing a nanoparadigm for hymenolepiasis tackling at lower dose levels. For consummating this goal, PRZ-PMMs were tailored *via* thin-film hydration technique integrating a binary system of Lutrol F127 and Gelucire 44/14. Box-Behnken design was planned for optimizing the nanoformulation variables employing Design-Expert^®^ software. Also, in *Hymenolepis nana*-infected rats, the pharmacodynamics of the optimal micellar formulation versus the analogous crude PRZ suspension were scrutinized on the 1st and 3rd days after administration of a single oral dose (12.5 or 25 mg/kg). Moreover, in vitro ovicidal activity of the monitored formulations was estimated utilizing Fuchsin vital stain. Furthermore, the in vivo pharmacokinetics were assessed in rats. The optimum PRZ-PMMs disclosed conciliation between thermodynamic and kinetic stability, high entrapment efficiency (86.29%), spherical nanosized morphology (15.18 nm), and controlled-release characteristics over 24 h (78.22%). ^1^H NMR studies verified PRZ assimilation within the micellar core. Additionally, the in vivo results highlighted a significant boosted efficacy of PRZ-PMMs manifested by fecal eggs output and worm burden reduction, which was clearly evident at the lesser PRZ dose, besides a reversed effect for the intestinal histological disruptions. At 50 µg/mL, PRZ-PMMs increased the percent of non-viable eggs to 100% versus 47% for crude PRZ, whilst shell destruction and loss of embryo were only clear with the applied nano-cargo. Moreover, superior bioavailability by 3.43-fold with elongated residence time was measured for PRZ-PMMs compared to PRZ suspension. Practically, our results unravel the potential of PRZ-PMMs as an oral promising tolerable lower dose nanoplatform for more competent PRZ mass chemotherapy.

## 1. Introduction

Intestinal parasitic ailments are a serious international health burden afflicting over a quarter of the global inhabitants provoking immense morbidity and mortality in underprivileged communities [1]. *Hymenolepis nana* (*H. nana*) cestode, typically recognized as the dwarf tapeworm, is the utmost prevalent intestinal helminth particularly in children arousing worldwide infection surpassing 75 million individuals [2]. Infection with *H. nana* in humans occurs *via* ingestion of contaminated foodstuffs with eggs or feces from infested humans/rodents. As well, it could be accidentally attained through arthropods ingestion such as fleas and flour beetles, which represent the intermediate hosts harboring cysticercoid stages with subsequent development into mature worms within the gastrointestinal tract (GIT) [3]. Furthermore, *H. nana* depicts the sole cestode directly transmitted from an individual to another besides frequent incidence of endogenous autoinfection, a hallmark that might evoke implications into the infection prognosis among patients [4]. *H. nana* infection, termed hymenolepiasis, may be asymptomatic, yet intense infections could bring about diarrhea, abdominal pain, gagging, vomiting, anorexia, anal itching as well as vague GIT manifestations [5]. Additionally, dizziness, headache and behavioral/sleep disturbances are iteratively narrated. Moreover, hymenolepiasis can be a life-threatening disseminated disease, chiefly among immunosuppressed individuals with HIV and malnourished pediatrics [6]. Hymenolepiosis diagnosis could be consummated through microscopic examination of stool samples for eggs or *via* adult cestodes detection in intestinal necropsy. Alongside, identification of cysticercoids within lamina propria of the intestinal villus should be salutary [7]. Currently, praziquantel (PRZ) is the mainstay of hymenolepiosis tackling administered as two separate doses of 25 mg/kg body weight with a lag interval of 10 days. The second dosage might mitigate the hazard of relapses [8].

PRZ is a heterocyclic pyrazinoisoquinoline employed as the cornerstone drug modality against diverse parasitic diseases occasioned by cestodes as well as trematodes in both human and veterinary medicine. Also, it is broadly utilized in hydatid management campaigns in addition to treatment of neurocysticercosis, clonorchiosis, opisthorchosis and schistosomiasis [9]. The PRZ mechanism of action is linked with spurring calcium influx with subsequent upsurge in its intracellular levels of helminthes. Its main molecular target might be proteins constituting calcium channels as well as donating calcium homeostasis. Muscular spastic paralysis of helminths and morphological modulations, particularly tegument disintegration, are noticeable manifestations of PRZ action [10]. Despite its clinical significance, PRZ elicits diminutive variable oral bioavailability pursuant to its sparse hydrosolubility, rigorous hepatic metabolism and fast circulatory clearance with miniaturized terminal half-life (1–1.5 h) [11]. The aforementioned encumbrances dictate recurrent high PRZ oral dose administration to warrant adequate plasma levels at larval tissues for the cestodal infection eradication. Additionally, PRZ treatment is primarily associated with adverse impacts such as giddiness, headache, nausea, vomiting, rash and diarrhea evidencing definite PRZ toxicity [10], and hence it is mandatory to reduce its therapeutic doses. In this quest, the engineering of nanomedicines has been vastly explored as a remarkable surrogate to ameliorate the cellular delivery and in vivo biofate of hydrophobic medications like PRZ in order to accomplish safe and effective doses. In particular, lipid-based nano-cargos comprising solid lipid nanoparticles [12], liposomes [13], niosomes [14] and nanocapsules [11] divulged consolidated PRZ bioavailability and antihelminthic activity. Herein, polymeric mixed micelles (PMMs) were refined as a promising nanoparadigm to overwhelm the redundant oral-related PRZ shortcomings.

PMMs are kinetically stable nanosystems tailored *via* the self-assembly of amphiphilic entities in water, beyond critical micelle concentration (CMC), into a core-shell architecture. The inner core constitutes the hydrophobic portion of the PMMs embedding lipophilic drugs such as PRZ, whereas the hydrophilic corona surmounts drug inactivation by biological milieu like the GIT [15]. A representative of these polymers is Lutrol block copolymers, which are versatile amphiphilic polymers composed of hydrophobic poly(propylene oxide) (PPO) as a central block flanked by two hydrophilic poly(ethylene oxide) (PEO) blocks basically constructed into a triblock array: PEO–PPO–PEO. Such block copolymers are FDA approved, biocompatible, readily obtainable and tolerable. Nevertheless, singular type Lutrol based-micelles are correlated with larger particulate size, inferior drug loading, as well as diminished stability [16]. Recently, mixed di/multifunctional micelles, assembled *via* sagacious amalgamation of various polymers, have triggered curiosity as auspicious nanovectors to surpass the negative features of monomicellar systems [17]. In this study, binary micellar nanoassemblies of Lutrol F127 (LF127: EO_100_–PO_65_–EO_100_, HLB 22) and Gelucire 44/14 (GL44: Lauroyl polyoxyl-32 glycerides, HLB 14) were elaborated for reinforcing drug loading efficacy and micellar stability. GL44 is a non-ionic lipidic surface active substance allied to generally regarded as safe excipients which consists of blends of mono/di/tri-esters of glycerol besides mono/di-esters of PEGs [18]. It is extensively harnessed for fabrication of solid dispersions, nanoparticles, and self-emulsifying drug delivery systems. Furthermore, it has a potential aptitude to boost the solubility of hydrophobic drugs *via* micellar formation [19].

Intriguingly, the prospective exploitation of PMMs as oral nano-cargos was verified through promoted bioavailability and pharmacodynamics of various drugs encompassing valsartan [20], efavirenz [15] and icaritin [16], owing to their unique and inherent characteristics such as controllable and minute size (<100 nm), sustained release of the laden drugs, along with superior hydrosolubility and intestinal permeability. According to the preceding remarks, we hypothesized that PRZ-PMMs could enhance the bioavailability/cellular delivery, conquer the rapid clearance, upgrade the dosage proportionality and minimize the inter-patient variability, thus lowering the overall recommended dose to be orally delivered. To the best of our knowledge, no clinical trial for oral PRZ-PMMs exploitation in hymenolepiosis burden tackling has been reported.

Consequently, in the current work, PRZ-PMMs were assembled adopting thin-film hydration method based on LF127/GL44. The modeling along with optimization of nanoreservoirs was pursued applying Box-Behnken statistical design. Also, the pharmacodynamic activity of the optimal PRZ-PMMs was assessed in *H. nana* infected rats one and three days post-administration of either 12.5 or 25 mg/kg as a single oral dose as compared with the correspondent oral PRZ suspension. Additionally, intestinal histopathological examinations as well as kidney and liver functions were estimated to prove the efficacy and rule out any negative effects of PRZ-PMMs. Furthermore, to substantiate the in vivo behavior, the pharmacokinetics of the tested formulations were scrutinized in male Wistar rats.

## 2. Materials and Methods

### 2.1. Materials

Praziquantel was acquired as a gift sample from Egyptian International Pharmaceuticals Industries Co. (EIPICO) (Cairo, Egypt). Gelucire 44/14 was kindly donated by Gattefossé (St-Priest, France). Lutrol F127, Tween 80, sodium lauryl sulfate, sodium carboxymethyl cellulose, methanol (HPLC grade), formic acid (HPLC grade) and methyl-*ter*-butyl ether (HPLC grade) were obtained from Sigma-Aldrich (St. Louis, MO, USA). Dialysis bags with a molecular weight cut off of 12,000 Da were procured from SERVA Electrophoresis GmbH (Heidelberg, Germany). All other chemicals and reagents were of analytical grade.

### 2.2. Experimental Design

In the current work, the software Design-Expert^®^ (Version 12.0.3.0, Stat-Ease Inc. Minneapolis, MN, USA) was utilized to construct a 15-run, three-level Box-Behnken design of three factors in order to optimize PRZ-PMMs. This design comprises three replicated central points and 12-sets of points bent upon the midpoint of each edge of the multidimensional cube which outlines the area of interest. Such cubic design is adequate for scrutinizing quadratic response surfaces as well as creating second-order polynomial models. According to preliminary screening, the causal factors elected for investigation were LF127 (*X*_1_), GL44 (*X*_2_) and PRZ (*X*_3_) concentrations, which are practically deemed as preeminent variables in the assembly of PMMs. These formulation moderators were explored at three distinct levels such as low (−1), medium (0) and high (+1), as denoted in Table 1. The dependent variables involved PRZ entrapment efficiency percent (*Y*_1_: EE%), micelle size (*Y*_2_) and accumulative % PRZ released over 24 h from PMMs (*Y*_3_: Q_24_). The recorded response *Y* could be forecasted from the following non-liner quadratic model equation:

(1) $$Y=b_{0}+b_{1}X_{1}+b_{2}X_{2}+b_{3}X_{3}+b_{12}X_{1}X_{2}+b_{13}X_{1}X_{3}+b_{23}X_{2}X_{3}+b_{11}X_{1}^{2}+b_{22}X_{2}^{2}+b_{33}X_{3}^{2}$$

where, $b_{0}$ is the intercept, $b_{1}$, $b_{2}$, $b_{3}$ are linear coefficients, $b_{12}$, $b_{13}$, $b_{23}$ are joint coefficients and $b_{11}$, $b_{22}$, $b_{33}$ are quadratic coefficients [21].

### 2.3. Fabrication of Self-Assembled PRZ-PMMs

PRZ-PMMs were tailored, embedding a combinatorial mixture of LF127/GL44, adopting thin-film hydration method [22]. In brief, accurately weighed amounts of PRZ, LF127 and GL44 were dissolved in chloroform (5 mL) in a rounded-bottom flask. Thereafter, the solvent was gradually evaporated under vacuum at 60 °C for 30 min under −60 Kpa pressure until a thin film of PRZ-copolymeric matrix was created on the flask wall of a RE300 Stuart rotary evaporator (Wolf Laboratories, North Yorkshire, UK) which was fixed with a RE3022C Stuart vacuum pump (Wolf Laboratories, North Yorkshire, UK). In a desiccator, the nascent film was held under vacuum for 2 h until solvent residues were totally eliminated. To ascertain full film hydration, the resultant dried-film was treated with 10 mL distilled water wherein the flask was revolved for 30 min at 60 rpm under normal pressure and elevated temperature (60 °C), so that a clear micellar dispersion was attained. Blank PMMs were fabricated utilizing an analogous procedure.

### 2.4. In Vitro Characterization of PRZ-PMMs

#### 2.4.1. Assessment of PRZ Content in PMMs

To assess the percent EE, drug-loading (DL), and precipitated drug (PD) of micelles, centrifugation of the assembled formulations was performed to detach PRZ-PMMs from the non-entrapped PRZ. This process was achieved by employing a SIGMA 3–30 K cooling centrifuge (Steinheim, Germany) adjusted at 14,000 rpm and 4 °C for 1 h. After adequate dilution of the transparent supernatant with ethanol, the concentration of entrapped PRZ was estimated, in three replicates, *via* a V-530 Jasco spectrophotometer (Jasco, Tokyo, Japan) tuned at 263 nm (λ_max_) [23]. The next set of equations was applied to compute EE, DL as well as PD percentages:

(2) $$\mathrm{EE}\%=\frac{\mathrm{amount}\;\mathrm{of}\;\mathrm{the}\;\mathrm{drug}\;\mathrm{in}\;\mathrm{PMMs}}{\mathrm{amount}\;\mathrm{of}\;\mathrm{the}\;\mathrm{feeding}\;\mathrm{drug}}\times 100$$

(3) $$\mathrm{DL}\%=\frac{\mathrm{amount}\;\mathrm{of}\;\mathrm{the}\;\mathrm{drug}\;\mathrm{in}\;\mathrm{PMMs}}{\mathrm{amount}\;\mathrm{of}\;\mathrm{the}\;\mathrm{feeding}\;\mathrm{polymer}\;\mathrm{and}\;\mathrm{drug}}\times 100$$

(4) $$\mathrm{PD}\%=\frac{\mathrm{drug}\;\mathrm{content}\;\mathrm{of}\;\mathrm{PMMs}\;-\;\mathrm{drug}\;\mathrm{content}\;\mathrm{of}\;\mathrm{PMMs}\;\mathrm{after}\;\mathrm{storage}}{\mathrm{drug}\;\mathrm{content}\;\mathrm{of}\;\mathrm{PMMs}}\times 100$$

Considering Equation (3), the drug content of PMMs denotes PRZ amount determined right after the fabrication of the micelles whereas the PRZ amount in PMMs following storage for 24 h at 4 °C was denoted as the drug content of PMMs after storage [24].

#### 2.4.2. Assessment of Micelle Size and ζ Potential

To appraise the average micellar size (z-ave) in addition to ζ potential and polydispersity index (PDI) of PRZ-PMMs, the dynamic light scattering (DLS) in a Nano ZS Zetasizer (Malvern instruments, Malvern, UK) was employed. For nullifying the multi-scattering issue, each specimen was diluted before the analysis by addition of distilled water. The analysis was executed at room temperature (25 ± 2 °C) and perpendicular angle of the incident beam (90°) after an equilibration time of 120 s [25]. Three scans were carried out on each specimen to narrate mean values ± SD.

#### 2.4.3. Assessment of PRZ-PMMs In Vitro Release Behavior

In triplicate, the release behavior of PRZ from the assembled PMMs was investigated utilizing a type 1, SR 8 Plus USP dissolution tester apparatus (Hanson Research, Chatsworth, Los Angeles, CA, USA) employing the membrane diffusion procedure [26]. With regard to the estimated EE%, specified volumes of PRZ-PMMs (equalizing 3 mg PRZ) were relocated into glass cylinders of 6-cm length and 2.5-cm internal diameter, which were neatly sealed from one ending with a presoaked dialyzing membrane (12,000 Da molecular weight cut off). For establishing the sink condition, 70 mL of phosphate-buffered saline (PBS) comprising 0.2% *w/v* sodium lauryl sulfate (SLS) was used as the release milieu after adjusting the pH to 7.4 and setting the temperature to 37 ± 0.5 °C [27]. After fixing the filled cylinders to the USP dissolution apparatus shafts, a revolving speed of 50 rpm was applied. About 1-mL aliquots were intermittently pulled out after 0.5, 1, 2, 4, 6, 8, 12 and lastly after 24 h as prescheduled time intervals. In order to maintain the volume of the dissolution milieu at a fixed level, each aspiration was recompensed with a fresh milieu. After applying the appropriate dilution, a spectrophotometer set at λ_max_ 263 was used to quantify PRZ in the collected samples. Afterwards, PRZ accumulative % released, as mean (±SD) values, was graphed versus time. An analogous experimentation was conducted on 2 mL of 1.5 mg/mL free PRZ aqueous suspension for comparative purposes. For assessing the release kinetics of PRZ-PMMs, the time course of % PRZ released was run through selected kinetic models, namely, zero-order, first-order, Higuchi, Korsmeyer–Peppas in addition to Hixson–Crowell models. Amplification of the coefficient of determination (R^2^) made the grounds for picking the most appropriate arithmetic model.

### 2.5. Optimization of PRZ-PMMs

Through adopting the desirability approach within the Design Expert^®^ software, the optimum nanoformulation was provided by employing constraints on EE% and Q_24_ for accomplishing the maximum estimates and on micelle size for attaining the miniaturized value. The ultimate goal was to get an optimal solution corresponding to a desirability index that is closest to unity. The recommended optimal nanoformulation was then assembled and examined thrice. The average values of the measured responses were then matched with the anticipated responses conferred by the software in order to check the validity of the composition of the designated optimized formulation.

### 2.6. Transmission Electron Microscopy (TEM)

The morphological features of the optimal PRZ-PMMs were inspected *via* a JEM-1400 transmission electron microscope (Jeol, Tokyo, Japan). After proper dilution of the micellar nanodispersion, a drop was introduced on a grid made of copper and a filter paper was used to expel the surplus. Subsequently, a negative stain composed of 2% *w*/*v* phosphotungstic acid aqueous solution was dropped, and likewise the excess was drawn out. Lastly, at ambient temperature, the air-dried sample was investigated, exploiting TEM at 80 kV [28].

### 2.7. Characterization through ^1^H NMR

After lyophilization of PRZ-PMMs employing a Novalyphe-NL 500 freeze-dryer (Savant Instruments, Holbrook, NY, USA), the drug-loading attributes of the micelles were inspected by recording ^1^H NMR spectra of PRZ, blank PMMs and PRZ-PMMs in deuterated chloroform (CDCl_3_) on a Bruker Avance III 400 MHz NMR spectrometer (Bruker AG, Zurich, Switzerland) at ambient temperature. For contrasting purposes, ^1^H NMR spectra of PRZ-PMMs were recorded in deuterated water (D_2_O) [29]. Chemical shifts were quoted in δ, allied to that of the solvents and determined from residual protons in CDCl_3_ or D_2_O. A lag interval of 15 min in every spectrum enabled a stabilized sample temperature prior to data acquisition [30].

### 2.8. Physical Stability Study of PRZ-PMMs

The optimized PRZ-PMMs nanoformulation was stored in locked glass vials at ambient temperature for 90 successive days in order to examine physical stability. Specimens from the micellar dispersion were pulled out right after assembly, then cyclically after 30, 60 and 90 days of storage. The aspirated specimens, filtrated *via* 0.22 µm membrane filter, were investigated for PRZ EE%, z-ave, ζ potential and Q_24_ throughout the storing period wherein determinations were iterated thrice. Furthermore, statistical differentiation amongst the explored release profiles of both fresh and stored PMMs was performed reliant on a model-independent arithmetical approach [31]. The similarity factor ($f_{2}$) was computed employing the following equation:

(5) $$f_{2}=50log\left\{{\left[1+\left(\frac{1}{n}\right)\overset{n}{\underset{t=1}{\sum }}{\left(R_{t}-T_{t}\right)}^{2}\right]}^{-0.5}\times 100\right\}\;$$

where n is the sampling number,$\;R_{t}$ and $T_{t}$ denote the average % released from the freshly prepared micellar formulation (reference) and the stored formulation (test) at time t, respectively. To declare congruity of the release profiles, an $f_{2}$ value ≥ 50 should be recorded.

### 2.9. In Vivo Study in H. nana-Infected Rats

#### 2.9.1. *H. nana* Collection

Mature *H. nana* worms were gathered from naturally-infected *Mus musculus* and kept in outbred rats. Thereafter, collection of the eggs was executed from infected rats that were euthanized on day 16th post infection. Infectious shell-free eggs were produced from the gravid segments of adult worms *via* agitating the egg suspension with 3 mm-diameter glass beads shortly prior to use [32]. 

#### 2.9.2. Animals and Experimental Design

Sixty Wistar rats, procured from the animal facility of the Research Institute of Ophthalmology (Giza, Egypt), were divided into six groups of 10 animals each. The rats were males weighing 120–150 g and were free of parasitic infections. The rats were housed in a specific pathogen-free environment under standard conditions (22 ± 2 °C and 50–60% humidity) and were supplied food and water *ad libitum*. Rats of groups I-V were inoculated with shell-free eggs *via* the oral route using a stomach tube. Each animal received 3000 eggs in 0.1 mL of PBS pH 7.4. At day 16th post infection, a fecal examination was carried out and egg count per gram was detected using the McMaster technique [33]. Group I was used as the infected non-treated control group. At the peak of egg production (20 days post infection), the treated groups received a single oral dose of PRZ as follows: groups II and III received 25 and 12.5 mg/kg of crude PRZ, respectively. Meanwhile, groups IV and V received 25 and 12.5 mg/kg of PRZ-PMMs, respectively. Animals of group VI were used as the non-infected non-treated control group. The study protocol and procedures were approved by the local Animal Care and Use Committee of Beni-Suef University (protocol approval code: REC-A-PhBSU-20015). All procedures for agent administration, blood and tissue manipulation were in accordance with the Guide for the Care and Use of Laboratory Animals (8th edition) published in 2011 by the United States National Academy of Sciences.

#### 2.9.3. In Vivo Activity of PRZ

##### Fecal Egg Reduction

Fecal egg count per gram (EPG) was estimated in the different groups on day 21 and 23 of the experiment. Fecal egg reduction percentage was calculated based on the formula [34]:

$$Fecal\;egg\;reduction\;percentage=\frac{\left(EPG\;before\;treatment-EPG\;post\;treatment\right)}{EPG\;before\;treatment}\times 100$$

##### Worm Reduction

On the 23rd day of the experiment, the rats were euthanized and the small intestines were removed. The small intestines were longitudinally dissected and the worm burdens were assessed.

##### Estimation of Biochemical Parameters

Blood samples were collected from all the rats on the 23rd day of the experiment. Serum alanine amino-transferase (ALT) and aspartate aminotransferase (AST) activities were measured [35]. Also, serum creatinine level was detected [36]. Additionally, blood urea level was checked by urease-glutamate dehydrogenase [37].

##### Histopathological Study

Intestines of rats from different groups were gathered and instantly fixed for 24 h in neutrally buffered 10% formalin. Wet tissue sections were dehydrated through a series of alcohols and embedded in paraffin. Serial 4 μm thick cuts were made on all paraffin blocks by rotatory microtome. Thereafter, the cuts were fixed on glass slides and the paraffin was removed. After rehydration, hematoxylin and eosin (H&E) stain was applied according to the method of Bancroft and Gamble [38] for histopathological evaluation using Olympus (model BX53) light microscopy.

### 2.10. In Vitro Ovicidal Activity

The activity of PRZ and PRZ-PMMs, against *H. nana* eggs, was investigated using Fuchsin vital stain [39,40]. In eppendorf tubes, 100 *H. nana* eggs freshly deposited from the control untreated rats, were suspended in saline and incubated at 25 °C for 15 min with 0, 5, 10, 25 and 50 µg/mL of PRZ or PRZ-PMMs. Three tubes for each concentration of each formulation were prepared. On clean glass slides, 100 µL of eggs of the different treatments were added and stained with 20 µL of freshly prepared Fuchsin for 10 min and examined under a light microscope.

### 2.11. Pharmacokinetic Investigations

#### 2.11.1. Administration of PRZ to Rats

Twelve male Wistar rats (120–150 g), procured from the animal facility of the Research Institute of Ophthalmology (Giza, Egypt), were arbitrarily allocated into two groups of six rats each. The polyacrylic cages, which the rats were lodged in, were maintained under controlled temperature (22 ± 2 °C) and relative humidity (55 ± 5%), in the presence of day and night cycled over 12 h intervals. The animals were provided with tap water *ad libitum* and standard forage till the night before dosing was commenced so that a 12 h starvation period was allowed. PRZ, in the form of aqueous suspension comprising sodium carboxymethyl cellulose (0.5% *w*/*v*) and in the form of optimum PRZ-PMMs, was orally dosed as 12.5 mg/kg to both groups using oral gavage. Blood samples (0.5 mL) from retro-orbital plexus were withdrawn into heparinized tubes under anesthesia at programmed time intervals for 4 h for PRZ suspension and 24 h for PRZ-PMMs. Next, centrifugation at 4000 rpm was directly applied to the tubes for 10 min in order to collect plasma. The isolated plasma samples were stored at −20 °C pending assay. The current study was reviewed and approved by our institutional Animal Ethics Committee of Beni-Suef University (protocol approval code: REC-A-PhBSU-20015).

#### 2.11.2. Conditions of Chromatographic Analysis

A liquid chromatography–tandem mass spectrometry (LC–MS/MS) method [41] was employed to assay the gathered plasma samples for PRZ. An Agilent eclipse C18 column (Agilent Technologies, Santa Clara, CA, USA) with the dimensions of 3.5 µm × 4.6 mm × 100 mm was employed for chromatographic separation utilizing the isocratic elution technique. The mobile phase, consisted of methanol and 0.1% formic acid (85:15, *v*/*v*), was delivered at a flow rate of 0.8 mL/min (split ratio 1/1). A sample injection volume of 10 µL was utilized. The temperatures of the column and autosampler oven were set to 40 °C and 15 °C, respectively. Electrospray ionization (ESI) in the positive mode was used for the operation of the mass spectrometer. Nitrogen was employed as the auxiliary and nebulizer gas. Source temperature, nebulizer gas, curtain gas and heater gas (i.e., the principal operation parameters) were adjusted at 500 °C, 60 psi, 25 psi and 60 psi, respectively. The ion spray voltage was tuned at 5500 V. Entrance potential, declustering potential, collision energy and collision exit potential (i.e., the compound parameters) were 10, 130, 15, 20 V for PRZ and 10, 91, 29, 12 V for diazepam as an internal standard (IS), respectively. Quantification was performed in the multiple reactions monitoring mode with *m/z* 313.18 → 203.10 for PRZ and *m/z* 285.31→193.00 for IS. Operation of the detector was at 1900 V. Analytical data processing was carried out through Analyst software (Version 1.4.2, Applied Biosystems, MDS SCIEX, Concord, ON, Canada).

#### 2.11.3. Samples Preparation for Analysis

A procedure based on liquid–liquid extraction was utilized. Briefly, to a plasma sample (100 µL), 10 µL of IS solution (50 ng/mL) was spiked and 1 mL of methyl-*ter*-butyl ether was added. The mixture was vortexed for 30 s to ensure homogeneity and then centrifugation at 3000 rpm was applied for 10 min. Into a conical glass tube, the transparent supernatant was transmitted and left at ambient temperature until the organic layer was entirely evaporated. The residues resulting from the evaporation process were then dissolved in the mobile phase and 10 µL of the solution was subsequently injected through the autosampler. Linearity was observed over a concentration span of 0.05–100 ng/mL (R^2^ = 0.9991). Plasma concentration as low as 0.03 ng/mL made the lower limit of quantification (LLOQ) for PRZ determination in rats. PRZ extraction recoveries in rat plasma were in the range of 98.94–100.86%. Additionally, the assay was accurate (>95%), precise (CV < 5) and selective for PRZ determination in rat plasma.

#### 2.11.4. Pharmacokinetic Parameter Estimation

The non-compartmental pharmacokinetic analysis function built into the Excel Add-In software, PK Solver, was employed to analyze the time course of the PRZ concentration in rat plasma for estimation of pharmacokinetic parameters [25,28]. The software assesses the maximal drug concentration (C_max_, ng/mL) and elapsed time to attain such concentration (T_max_, h) directly from the plasma concentration–time curve. The software adopts the trapezoidal rule method for quantifying the area under the curve (AUC) from 0 to t (AUC_0–t_, ng h/mL) and from 0 to infinity (AUC_0–∞_, ng h/mL). Ultimately, relative bioavailability (F_rel_, %) of oral PRZ-PMMs was computed considering oral PRZ suspension as a standard.

### 2.12. Statistical Analysis

Verifying normality of the distribution of the response variables was achieved through the Kolmogorov–Smirnov test. All continuous data were enumerated as mean ± SD. For normal quantitative variables, *t*-test or one-way ANOVA (with Duncan post-hoc test for subsequent multiple comparisons) were pursued for comparing two or more groups, respectively. The level of significance was set at *p* < 0.05. The computer program SPSS 22 (SPSS, Chicago, IL, USA) was utilized to accomplish all calculations.

## 3. Results and Discussion

PMMs elicit diverse intriguing traits as nanovectors involving their tunable composition, miniaturized size, facile assemblage and reduced cytotoxicity. Presumably, micelles are engineered *via* a reversible aggregating process where maintenance of the appropriate HLB of the nanosystem is pivotal for their stability. Greater system hydrophobicity curtails PMMs formation, whereas elevated hydrophilicity destabilizes the micellar system; hence, election of the optimum polymeric levels is key for kinetically and thermodynamically stabilized nanodispersions [22]. In the current work, we attempted to fabricate optically clear, small-sized and stable PRZ-PMMs based on LF127/GL44 without external energy processing, which could significantly consolidate the solubility of the sparsely-soluble PRZ in aqueous milieu.

### 3.1. Box-Behnken Design Analysis

**Figure 1:**
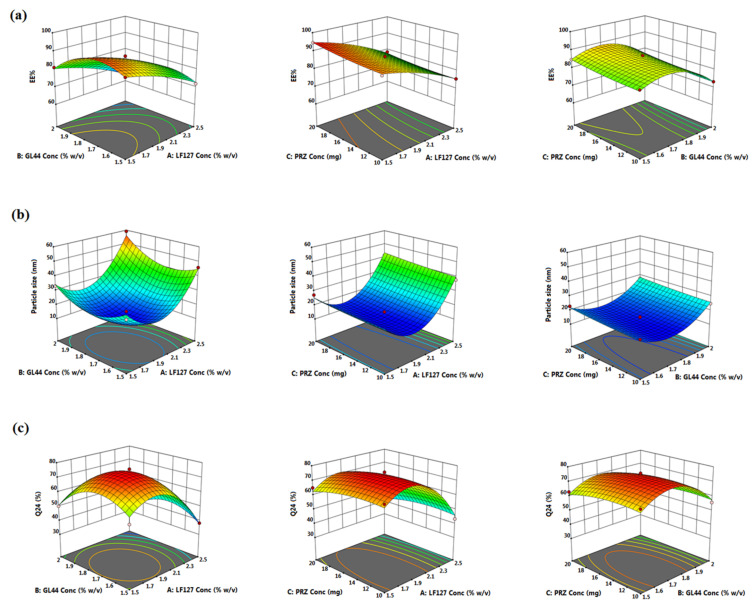
3D surface plots for the impact of LF127 (*X*_1_), GL44 (*X*_2_) and PRZ (*X*_3_) concentrations on (**a**) EE% (*Y*_1_), (**b**) micelle size (*Y*_2_) and (**c**) Q_24_ (*Y*_3_).

**Figure 2:**
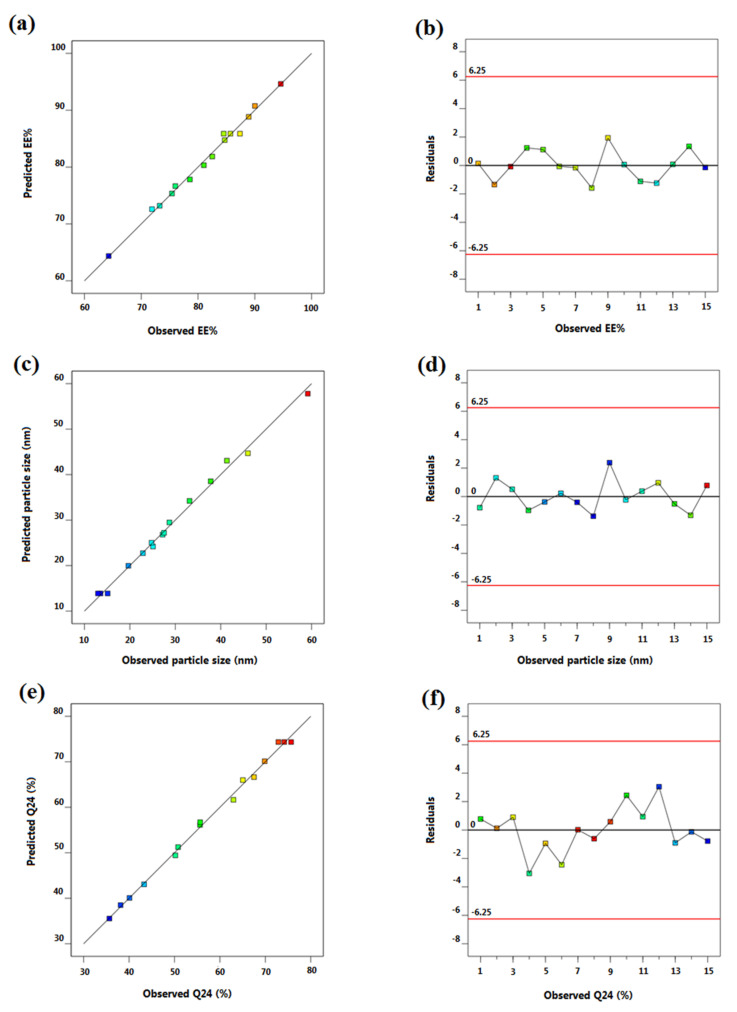
Linear correlation plots (**a**,**c**,**e**) between actual and predicted values and the correspondent residual plots (**b**,**d**,**f**) for different responses.

Experimental designing has been harnessed as a robust approach to mitigate the process variation, meanwhile conferring plenteous merits like accuracy, precision and prognosis [28]. Amongst diverse designing approaches, Box-Behnken design was utilized for optimization and analysis of main impacts, joint impacts as well as quadratic impacts of the process multivariable [21]. It is an incomplete three-factor three-level factorial design based on rotatable or approximately-rotatable second-order designs. Applying Box-Behnken design, the sample size is confined to a value essential for coefficients evaluation in a second degree least squares nearly polynomial form. A noteworthy feature of Box-Behnken design is that it abrogates experimental trials executed within extreme circumstances and does not simultaneously implicate combinations through which entire variables are at their minimal or maximal levels [42]. Herein, Box-Behnken design yielded 15 empirical runs for the fabrication of PRZ-PMMs with triple checkpoints as outlined in Table 2. The assembled formulations were scrutinized for EE% (*Y*_1_), micelle size (*Y*_2_) and Q_24h_ (*Y*_3_) as dependent responses. The estimation of signal-to-noise ratio is warranted to verify model adequacy to cruise through the space outlined by the design [43]. In all analyzed responses, the recommendable ratio of four and beyond was remarked, Table 3. Alternatively, prognosticated R^2^ was recorded for speculation of model integrity to forecast the value of the modeled response [44]. The algorithmic variance between the adjusted and prognosticated R^2^ values should be around 0.2 to be in a sensible agreement [45]. It is worth mentioning that the values of the prognosticated and adjusted R^2^ were harmonious in the three measured responses. Notably, too small coefficient of variation was noticed for the polynomial quadratic model in all the three responses emphasizing the model aptitude for exploring the design space. Analysis of the regression models of the observed responses spanning adequate precision, R^2^, SD as well as %CV are compiled in Table 3. Additionally, 3D response surface graphs overtly display the effects of causal factors on the recorded responses as per the Box-Behnken design, Figure 1. Furthermore, Figure 2 quantitatively correlates the actual versus prognosticated values for the various responses besides their residual plots. 

### 3.2. PRZ-PMMs Characterization

#### 3.2.1. Influence of Causal Factors on EE% (*Y*_1_)

The propensity of the assembled PMMs to ensnare considerable amounts of PRZ is crucial for their targeted exploitation for oral hymenolepiasis tackling. EE% is the percentage of the measured drug quantity trapped in PMMs with respect to the initial laded drug mass. PMMs flaunt with boosted encapsulation especially for hydrophobic moieties as PRZ with EE% values spanning from 64.27 ± 2.67 to 94.58 ± 4.05% at different levels of the three investigated causal factors as profiled in Table 2. Such infallible loading could offer an eligible maneuver for dose-associated toxicity through the enabling of lesser doses with escalated therapeutic efficacy [46]. Indeed, the loading of poorly-soluble drugs within micellar systems might be ascribable to either physical entanglement or chemical conjugation. Physical encapsulation of lipophilic entities by micelles is primarily achieved *via* forces of a different nature, namely Van der Waals forces, hydrophobic forces and hydrogen bonding [47]. In this work, the aromatic ring, carbonyl and tertiary amino functional groups of the active PRZ might be engaged in the hydrophobic interactions/hydrogen bonding with the micellar nanosystem. ANOVA evaluation of the perceived EE% values revealed best-fitting with the quadratic model reliant on its greatest R^2^ value. The F-value estimated for the model was 464.78, revealing the statistically significant impact of the causal factors (*p* < 0.0001) as summarized in Table 3. Additionally, lack of fit insignificantly differed from the pure error derived from the center replicates (*p* = 0.7315). Equation (6) denotes the quantitative impact of the three independent variables on the EE% of PRZ-PMMs (*Y*_1_) in terms of coded values:

(6) $$EE\%=+85.88-8.05X_{1}-4.19X_{2}+1.58X_{3}+0.07X_{1}X_{2}-0.35X_{1}X_{3}+0.14X_{2}X_{3}-1.91X_{1}^{2}-7.43X_{2}^{2}+0.68X_{3}^{2}$$

As per the regression coefficients, both LF127 (*X*_1_) and GL44 (*X*_2_) concentrations disclosed a marked negative impact on PRZ EE% values, whilst a significant positive influence was evinced by PRZ concentration (*X*_3_), *p* < 0.0001. Obviously, LF127 concentration (*X*_1_) exerted a more prominent impact on PRZ EE%. Figure 1a represents the response surface 3D plot for the joint effect of two causal factors on the average EE% values at the intermediate level of the third factor.

With respect to LF127 concentration (*X*_1_), the discernible higher EE% at lower copolymeric levels might be succumbed to the presence of hydrophilic EO chains at a lesser number, which spurs assimilation of the sparsely-water soluble PRZ. Conversely, destabilization of the micellar nanodispersions, owed to amplified system hydrophilicity, could be inaugurated upon raising LF127 concentration. Taken together, at elevated levels of LF127, disability of PRZ-PMMs to conserve shell integrity might trigger the compromised payload of PRZ within the micellar core [22]. These findings are in line with that declared by Patil et al. [48], who narrated an antagonistic influence of LF127 on the EE% of curcumin-PMMs.

As established from the statistical analysis conducted on EE% data, upraising GL44 (*X*_2_) concentration from 1.5 to 1.75% *w*/*v* was escorted with a significant snowballing in the mean EE%, whereas the EE% values of PRZ were dramatically declined with the further increment in GL44 concentration to 2% *w*/*v* (*p* < 0.0001). A hypothetical elucidation for the initial simultaneous upsurge in EE%, at middle GL44 levels, might be assigned to GL44-correlated hydrophobicity, which could have expedited PRZ partitioning into the hydrophobic micellar core. Controversially, the higher levels of GL44 might have destabilized the micellar system as a consequence of imbalanced state of attraction forces of core (lauric acids moieties, triglycerides) and repulsive forces existed through the larger PEG domains of GL44 in the exterior corona of micelles [18]. Remarkably, GL44 comprises 72% PEG with an HLB value of 14, hence once PEG is beneath the threshold, PRZ-PMMs should elicit adequate stability. Furthermore, GL44 molecules experience self-aggregation at higher levels, which could give a share in the dropping of PRZ EE% [49]. Such results are endorsed with prior literature arts [20,47,48]. 

Contrarily, PRZ concentration (*X*_3_) divulged a significant synergistic impact on the EE% of the tailored PMMs, *p* < 0.0001. This behavior might be interpreted in the notion of augmented hydrophobic interactions amongst drug and LF127/GL44 copolymers which surpassed those between PRZ molecules per se owing to the greater solubilization propensity of the copolymers as PRZ amount increased [23]. Wang et al. [50] reported comparable outcomes in their study on the assembly of difunctional Lutrols P105/L101 micellar nano-cargo for tumor targeting of paclitaxel.

Of note, the DL% of the developed PRZ-PMMs oscillated between 1.73 ± 0.16 and 5.48 ± 0.32% as recorded in Table 4. These lower values could be accredited to the integration of high PRZ: LF127/GL44 ratios; 1:16.25/42.5. These results are in a plausible harmony with those presented by Abdelbary and Tadros [51] who assumed that at an olanzapine:Lutrol mixture (L121:P123; 1:4) ratio of 1:40, the EE% and DL% were 75.03% and 1.84%, respectively. In our study, though the DL% was not high, PRZ solubility was strikingly upgraded to several times its intrinsic aqueous solubility limit. It is of interest to note that at a DL% of 5.48%, the effective PRZ level was 37.84 mg/mL, which was approximately 126.13-fold superior than its intrinsic hydrosolubility of ~0.3 mg/mL [10].

Following storage for 24 h at 4 °C, PMMs would attain equilibrium and the surplus PRZ should be deposited [24]. The PD% values of all PRZ-PMMs formulations fluctuated from 0.10 ± 0.02 to 0.66 ± 0.03% clarifying the potential of LF127/GL44 to create efficacious micellar nano-cargo with robust stability coupled with their small-sized assembly, Table 4. Also, such smaller PD% manifested could shed light on the assumption that PMMs would promote the hydrosolubility of lipophilic drugs alongside the lack of drug supersaturation since PRZ dosage was not exceeding the loading capacity of the developed PMMs.

#### 3.2.2. Influence of Causal Factors on Micelle Size (*Y*_2_)

Actually, vastly small-sized micelles of less than 100 nm with hydrophilic corona exhibit longer in vivo circulatory time. Such nano-cargo might monitor the drug administration rate, extend the therapeutic impact duration as well as promote the drug targeting potential to specific tissues. On the contrary, micellar sizes beyond 100 nm would display restrained biodistribution due to intensified capture *via* phagocytic cells as Kupffer cells within the reticuloendothelial system [52]. In the present study, the mean micelle size of PRZ-PMMs extended from 13.54 ± 2.33 to 59.17 ± 9.82 nm, signifying the assemblage of small-sized micelles, Table 2. For accomplishing the ANOVA presumption, the Box–Cox plot of micelle size proposed logarithmic response transformation. The investigated quadratic model following transformation was statistically significant in lieu of residual analysis and ANOVA with an adequacy/precision ratio of 64.42 delineating an adequate signal. The transformed regression equation interrelating the response variability to the three independent factors in coded values was:

(7) $$Ln\left(micelle\;size\right)=+2.63+0.24X_{1}+0.11X_{2}+0.05X_{3}+0.03X_{1}X_{2}+0.01X_{1}X_{3}-0.02X_{2}X_{3}+0.69X_{1}^{2}+0.38X_{2}^{2}+0.16X_{3}^{2}$$

The explored causal factors elucidated a pronounced influence on the micelle size amongst the various runs (*p* < 0.0001). As elucidated in Figure 1b, the average size of PRZ-PMMs at either lower or higher LF127 concentration levels was significantly larger as compared to its intermediate concentrations, *p* < 0.0001. A plausible explanation for the manifested micelle size shrinking might be the coexistence of an adequate number of hydrophilic EO domains, at the middle LF127 level, which could hamper secondary micellar clustering through steric hindrance [53]. Earlier reports claimed that impregnation of the extremely hydrophilic LF127 (EO_100_–PO_65_–EO_100_), possessing elongated EO groups, to the lipophilic copolymer Lutrol L121 (EO_5_–PO_68_–EO_5_) provoked fabrication of miniaturized spherical PMMs with reinforced kinetic stability [22,51,53]. Contrariwise, further increment in LF127 concentration was concomitant with a pronounced growth in the micellar size which might be attributed to inflation of micelles corona (PEO block of LF127) and/or elevated nanosystem hydrophilicity, which could have consequently destabilized PRZ-PMMs, triggering their aggregation [54].

Likewise, GL44 concentration (*X*_2_) exerted a biphasic influence on the mean micelle size of the tailored PRZ-PMMs (*p* < 0.0001), Figure 1b. Initially, the increment in GL44 level resulted in a synchronous reduction in the micellar size which might be inferred from stronger hydrophobic interactions among PPO domains of LF127 and the hydrophobic moiety of GL44 within the micellar core and/or diminution of interfacial tension *via* GL44 surface active characteristics lowering surface free energy with ultimate generation of compact micelles [49]. Furthermore, the enlarging tendency of micellar size with the subsequent increment in GL44 concentration could be imputed to distension of micellar corona due to the amplified number of PEG domains of GL44 relative to its hydrophobic portion [55]. These data are parallel to that accomplished by former investigations [18,20,48].

With regard to PRZ concentration (*X*_3_), it had a positive unfavorable impact on the size of PMMs (*p* < 0.0001). This observation could reflect a definite expansion of the hydrophobic micellar core size as a result of PRZ solubilization there. Virtually, this result is not surprising if it is elucidated on the basis of EE% values, wherein raised PRZ concentrations were accompanied with higher PRZ trapped amounts into the micelles and thus larger PMMs were attainable. This observation coincides with that proposed by Wei et al. [23], who prepared paclitaxel-laden Lutrols P123/F127 PMMs based on Doehlert matrix design.

Concerning PDI, it represents a dimensionless number that denotes the distribution of particulate size of the scrutinized nanosystem. The closer the PDI values to zero, the more uniform are the micellar population [25]. The PDI of the fabricated PRZ-PMMs vacillated between 0.12 and 0.45, reflecting narrowed-size distribution besides prominent homogeneity, Table 4.

#### 3.2.3. Influence of Causal Factors on Q_24_ (*Y*_3_)

The accumulative release pattern of free PRZ and PRZ-PMMs, in PBS pH 7.4 under sink condition at 37 °C, is graphically clarified in Figure S1a,b (Supplemental File). PRZ-PMMs were slowly diffused to the release milieu where the Q_24_ from the different formulations varied in the range of 35.67 ± 1.62 to 75.67 ± 4.79% compared with the significantly greater rate of 98.81 ± 5.94% for crude PRZ at 1 h pointing out dialyzability, *p* < 0.05. For non-polar drugs, fulfilling the sink condition is one of the obstacles for executing the in vitro release experimentations. In the present work, the sink condition was established *via* the incorporation of 0.2% *w*/*v* SLS in PBS with periodic replenishing of fresh milieu throughout the release course [27]. To ascertain delivery of the laden poorly-soluble drug to its absorptive site, the tailored nano-cargo should have the capability to battle fast dissociation with dilution and suppression *via* the harsh GIT conditions. Otherwise, release of the drug at a rapid pace could hasten a preceding sedimentation in the GIT aqueous milieu prior to absorption [56]. Hence, the salient sustained release of PRZ from LF127/GL44 PMMs might abrogate the quick precipitation and seepage into the GIT lumen implying conformational integrity of the assembled micelles, which is substantial for drug delivery and plasma/lymphatic micellar circulation. Similar findings for icariside II [57] and oridonin [58] release from Solutol HS15/F127- and Soluplus/P105-based PMMs, respectively, were already reported. In general, PRZ efflux from various PMMs was ostensibly biphasic process with a comparatively slight burst release during the earlier phase (first 2 h) supervened by a sustained release behavior over the empirical time. Such pattern of release might be clarified *via* PRZ geometrical destination within the micelles and highlighted the PRZ integration stability. The initial burst could be mostly allocated to the hydrophilic micellar corona-entangled drug and might progress through hydration of the superficial drug moieties combined with their passive diffusion. Afterwards, the delayed PRZ release stemmed from the molecules residing within the micellar core [59]. Kulthe et al. [60] reported the rigorous clasping of lipophilic drugs in the interior hydrophobic micellar core ensuing highly retarded drug release rates despite accomplishing the in vitro sink condition.

A quadratic correlation between the three causal factors and the Q_24_ response (*Y*_3_) was manifested with adjusted R^2^ of 0.9946, demarcating that nearly 99% of the net variations in the response would be described through this model. A Box–Cox plot of the Q_24_ suggested power transformation with lambda = −1.65 for warranting adequacy of the model. Therefore, the Q_24_ would be allied to the three causal variables *via* the subsequent equation in terms of coded values:

(8) $${\left(Q_{24}\right)}^{-1.65}=+0.0008+0.0006X_{1}+0.0002X_{2}+0.0008X_{1}^{2}+0.0004X_{2}^{2}$$

Statistical examination of the release data elucidated significant antagonistic influences of both LF127 (*X*_1_) and GL44 (*X*_2_) concentrations on the Q_24_ of PRZ-PMMs (*p* < 0.0001), Figure 1c. Though raising PRZ concentration (*X*_3_) gave rise to a higher burst effect with an overall negative impact on the Q_24_, such inflection was demonstrated to be statistically insignificant (*p* = 0.1944).

Parallelly, the initial increment in both LF127 and GL44 concentrations was linked with accentuated PRZ release rates. This phenomenon could be attributed to the hydrophilic trait of both LF127 (PEO domains) and GL44 (PEG chains), which promoted water permeability as well as PRZ diffusion across the copolymeric matrices bringing about the creation of additional hydrophilic channels with subsequent hastened drug release rates [23]. In addition, the minute size of such nanoreservoirs could donate a greater surface area/volume ratio exposed to the release milieu. In contrast, sluggish PRZ release rates were detectable upon further increment in LF127/GL44 levels, which could account for swelling of the outer micellar shells causing their agglomeration, as outlined before, besides their relatively magnified micellar size. Furthermore, the current findings illuminated dependency of PRZ release on micellar size.

The release data of PRZ-PMMs were fitted to sets of models attempting to anticipate the drug release mechanism. Table S1 (Supplemental File) outlines the computed regression-linearity coefficient (R^2^) for the recommended release kinetic models. Surprisingly, various releasing models were perceived since the elaborated micellar formulations revealed zero-, first-, as well as Higuchi-release kinetics. These findings might refer to diverse processes monitoring the liberation of the laden drug from PMMs comprising swelling/erosion, diffusion across the copolymeric matrix, and the degradation of the polymer to its monomers [46]. These mechanisms may occur solitarily or combined in a specified releasing system.

Additionally, Korsemeyer-Peppas model was adopted and the release exponent (*n*) amongst different dispersions ranged from 0.55 to 0.73. According to these values (0.5 < *n* < 1), PRZ release could be *via* the non-Fickian (anomalous) pattern in which the hybridization of molecular diffusion with copolymer chain relaxation/erosion might take place [28,42].

### 3.3. Identification of PMMs Optimal Composition

Generally, the optimization of pharmaceutical nano-cargos is pursued to assign the optimized levels of causal variables necessitated for fulfilling a highly qualified product with optimum physicochemical aspects [61]. Hence, a desirability function approach was exploited for electing the optimal composition of nanoformulation from the assembled 15 PMMs based on the applied 3^3^ Box-Behnken design. The constraints imposed to get the optimum micellar dispersion (maximal EE% and Q_24_ as well as minimal z-ave) were spanned, achieving an overall desirability index close to unity (0.919). The optimized formulation was tailored harnessing 1.79% *w*/*v* LF127, 1.68% *w*/*v* GL44 alongside 15.85 mg PRZ and displayed EE% of 86.29 ± 3.26%, z-ave of 15.18 ± 2.93 nm and Q_24_ of 78.22 ± 4.01%. As marked in Table 5, the actual values of the optimum nanoformulation were well concurred with the anticipated ones, reflecting a slight % prediction error which was in the range of 3.96 to 5.86% for the monitored responses, underlining the lack of curvature as well as validity/adequacy of the opted arithmetical models to assess the explored responses. Thus, the above composition was picked to formulate the optimal PRZ-PMMs for further analyses. 

### 3.4. Transmission Electron Microscopy

**Figure 3:**
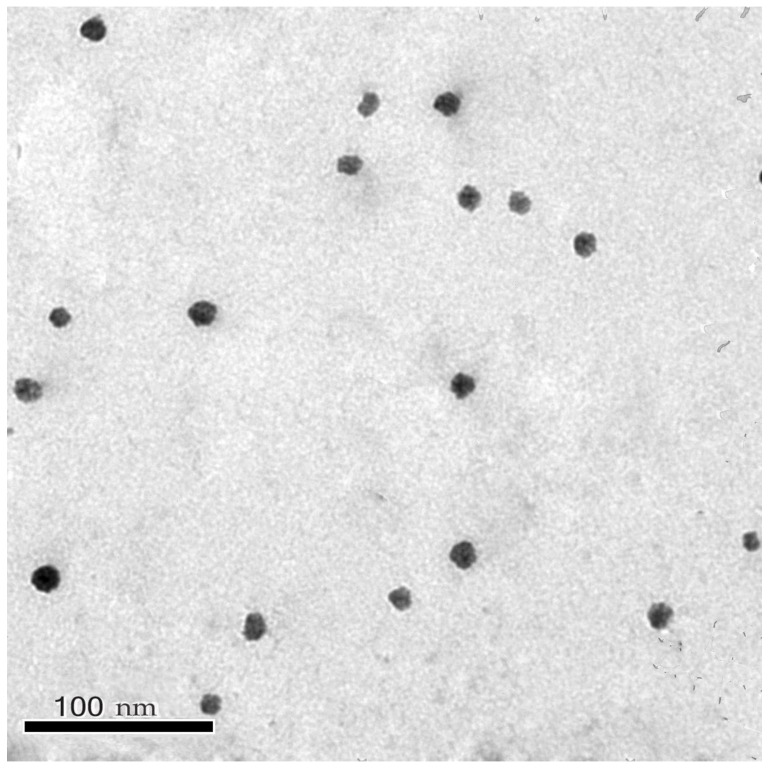
Transmission electron photomicrograph of the optimized PRZ-PMMs.

A representative photomicrograph of the air-dried optimal PMMs formulation demonstrated that the self-assembled micelles were clearly discrete, spherically-shaped and uniformly distributed in the size range of 12.08–16.23 nm, Figure 3, which emphasizes the competency of the nano-cargo to bypass systemic devastation and opsonization *via* circulating phagocytes [52]. Additionally, morphological examination was congruent with the mean micellar size recorded *via* DLS. Moreover, TEM imagining elucidated the core-shell structuring of LF127/GL44 PMMs, appearing as bright corona and dark core regions, which is very crucial for conferring extended plasma circulation time [20].

### 3.5. ^1^H NMR Characterization

**Figure 4:**
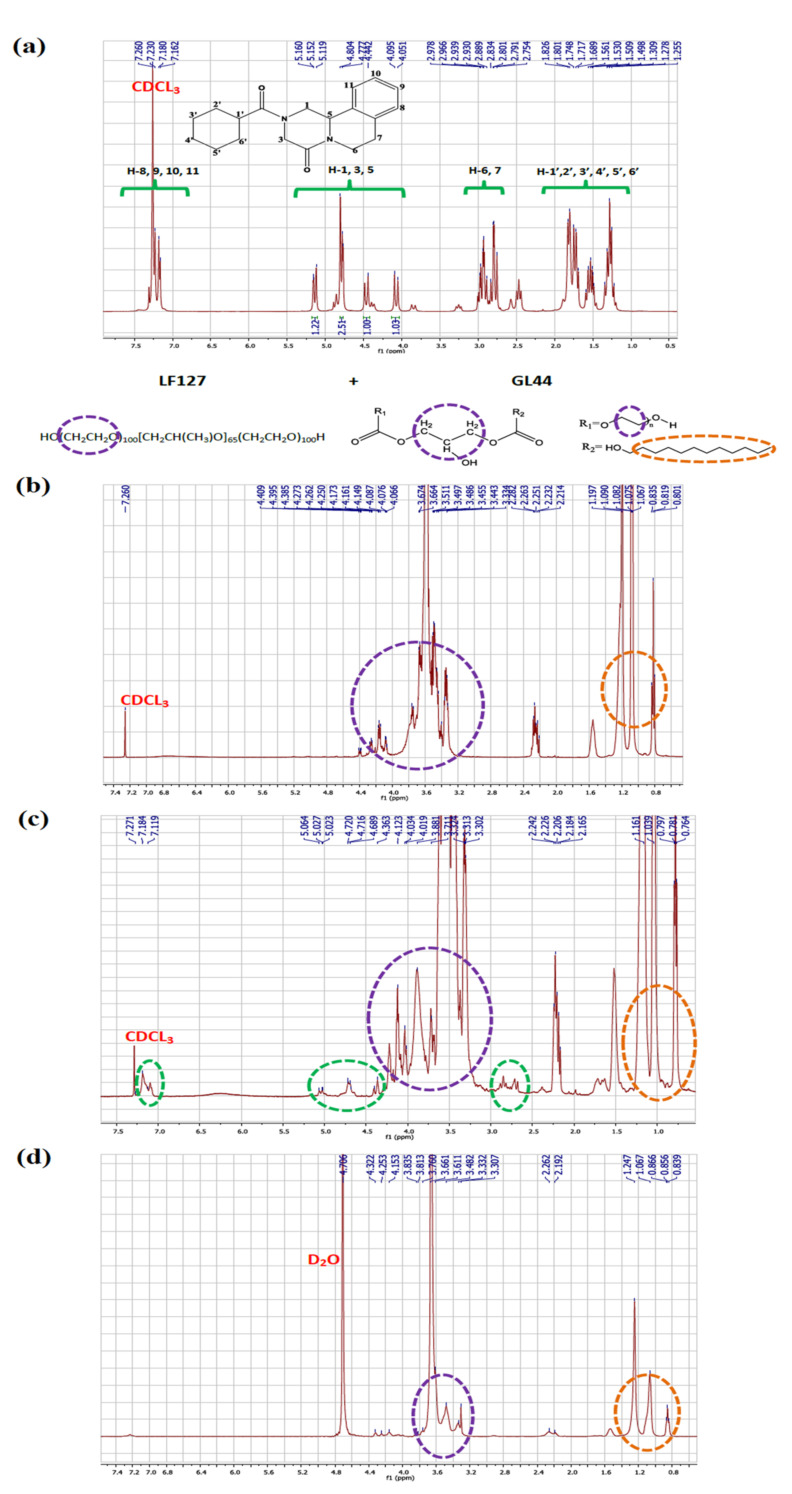
^1^H NMR spectra of (**a**) PRZ in CDCl_3_, (**b**) blank PMMs, (**c**) PRZ-PMMs in CDCl_3_ and (**d**) PRZ-PMMs in D_2_O with peaks allocated according to the corresponding molecular structures.

PRZ entrapment within the interior core of LF127/GL44 PMMs was ascertained through the investigation of ^1^H NMR spectra. Figure 4 displays PRZ, blank PMMs, and PRZ-PMMs ^1^H NMR spectra in CDCl_3_ besides the spectrum of PRZ-PMMs in D_2_O. The resonance peaks are assigned in consistency with the analogous molecular configurations. In CDCl_3_, the characteristic peaks correspondent to PRZ and LF127/GL44 were distinctly identified. Conversely, only LF127/GL44 resonance peaks were observed in D_2_O whilst those of PRZ were receded. These results could denote a confined PRZ molecular displacement proposing a state of trapping and solubilization of PRZ within the internal hydrophobic core of the micelles. As previously introduced [29], such realizations are consistent with an assemblage of core/shell micellar-type in aqueous milieu. The present observations are in accordance with ^1^H NMR inspections of valsartan- and olanzapine-based PMMs in D_2_O [20,51].

### 3.6. Physical Stability Study of PRZ-PMMs

By the end of three months storage duration at ambient temperature, there was no observed layer separation or turbidity in the optimal PRZ-PMMs. Statistical examination revealed insignificant variation (*p* > 0.05) of the EE%, micelle size, ζ potential and Q_24_ of the stored micelles as contrasted with the fresh ones, Table 6. Additionally, both the fresh and stored micelles divulged comparable release profiles, which was evidenced *via* the computed similarity factor value ($f_{2}$ = 68.42), demonstrating no marked impact on drug release at the defined storage conditions. Moreover, ζ potential of −10.35 ± 1.68 mv, which is far lower than 25 mv proposing stability of the scrutinized micelles with lesser aggregation probability, is chiefly owed to the steric influence on the micellar surface rather than the repelling forces among micelles [62,63]. Earlier studies proclaimed the pivotal role of LF127, due to its elongated PEO block, in nanosytems stabilization as a result of mitigating secondary micellar agglutination through steric hindrance [20,51,53]. Furthermore, GL44 functioned as a stabilizer, accentuating LF127-miceller stability. In particular, the boosted kinetic stability of PRZ-PMMs in aqueous milieu ensured the adequacy of LF127/GL44 amalgamation in the micellar nano-cargo.

### 3.7. In Vivo Study in H. nana-Infected Rats

A significant worldwide health burden is caused by parasitic flatworm diseases. PRZ oral treatment in tablet form is the primary drug therapy for treating these illnesses, according to the World Health Organization’s recently released roadmap for such infections over the period 2021 to 2030. Unluckily, the use of PRZ is restricted by a number of issues, including the high therapeutic dose required because of the drug’s poor solubility and bioavailability [64]. It may be possible to overwhelm PRZ drawbacks, enhance its efficacy and further save the enormous potential cost by incorporating it into a nanosized drug delivery system that can regulate its release at a therapeutically ideal rate and dose [65,66].

#### 3.7.1. In Vivo Egg Count and Worm Reduction Percentage

As depicted in Table 7 and Table 8, all of the treated groups showed significant reduction in EPG counts and worm burden (*p* < 0.001) compared to the infected control. Additionally, a significant difference was found between the pre- and post-treatment EPG counts. Treatments with crude PRZ 25 mg/kg and PRZ-PMMs 12.5 and 25 mg/kg significantly reduced the number of adult worms in the intestine and eggs output in feces by 100% compared to the infected control and crude PRZ 12.5 mg/kg treated group. Treatment with crude PRZ 12.5 mg/kg reduced the number of adult worms by 73.3% and eggs output in feces by 90.6 and 91.1% on the 21^st^ and 23^rd^ days, respectively. On the other hand, treatment with PRZ-PMMs 12.5 mg/kg increased the percent of reduction of adult worms and eggs output (100%). Similar to our results, Abou Shady et al. [39] assessed the effect of PRZ administered at 25 mg/kg dose on *H. nana*-infected mice. PRZ provoked a significant decline of the viable egg count, total egg output and worm burden. Another study conducted by Rashed et al. [67] evaluated the antihelminthic efficacy of PRZ against *H. nana* egg viability. Regarding the influence of PRZ on the number of viable eggs, there was a significant difference between the PRZ-treated group and the control group (*p* = 0.036). In a relevant study, Campos et al. [68] narrated full PRZ efficacy given at a dose of 25 mg/kg against adult *H. nana*-infected mice. These findings were achieved on days 10th and 14th post infection. This was clarified by Coles [69], who claimed the aptitude of PRZ to target constituents of aerobic respiratory pathways which are crucial not only for reproduction in both females and males but also for egg production, or it could be owed to the entire eradication of adult worms. 

The current art was concerned with fortifying the biological traits of the already-exploited antihymenolepis PRZ through assembly of PMMs as an innovative nano-cargo for upraising its bioavailability and subsequently tackling burdens to its biological activity at lower dose levels. In this work, significant boosted efficacy was recorded in *H. nana*-infected rats treated with PRZ-PMMs than those treated with the analogous crude PRZ suspension as mentioned above. Thus, a lower PRZ dose was adequate to achieve 100% killing of worms; PRZ-PMMs dose was reduced by 2-fold (12.5 mg/kg compared to 25 mg/kg for conventional PRZ). This efficacy could be related to the aptitude of PRZ-PMMs to reach the target sites in the organism, rapid solubility and potential improved bioavailability of PRZ with reduction of its toxicity [70]. Previous studies revealed higher PRZ efficiency upon its incorporation into a delivery carrier as montmorillonite clay against *S. mansoni*-infected mice, with a significant decrement in the dose by nearly 3-fold the traditional PRZ dose [66]. Also, compared with crude PRZ, PRZ-solid dispersion lessened the dose required to accomplish 50% killing of *S. mansoni* worms by 2-fold [27]. Additionally, PRZ-loaded solid lipid nanoparticles (SLNs) have presented fruitful outcomes over crude PRZ in cestodal infection at a lower dose (5 mg/kg) in dogs infected with *Echinococcus granulosus* [71]. Moreover, the decline in egg load was perceived in PRZ-SLNs-treated mice concomitant with entire absence of immature eggs, significant decrement in mature eggs and marked upsurge in dead eggs [72]. Furthermore, utilizing nanoprecipitation, PRZ nanosuspension elicited superior efficiency versus the cysticerci of the cestode parasite *Taenia crassiceps* [73].

#### 3.7.2. Biochemical Markers Analysis

Liver and kidney function tests of different studied groups are recorded in Table 9. Regarding liver enzymes, there was insignificant difference in ALT and AST activities in all treated groups compared with either normal control or with each other. With respect to kidney function, there were significant increases in both creatinine and urea levels in rats treated with crude PRZ (25 mg/kg) compared with normal control group. Additionally, there were significant declines in creatinine levels in rats treated with PRZ-PMMs at 12.5 and 25 mg/kg compared to rats administered crude PRZ (25 mg/kg), while urea levels were significantly reduced in rats received crude PRZ (12.5 mg/kg) or PRZ-PMMs (12.5 and 25 mg/kg) compared to rats treated with crude PRZ (25 mg/kg), Table 9. It is worth mentioning that creatinine and urea levels remained within normal limits in all treated groups. Collectively, administration of PRZ did not affect either liver or kidney functions as proved in the current study, which warrants safety and tolerability of treatment with both crude PRZ and PRZ-PMMs formulations with emphasis on superiority of PRZ-PMMs to crude PRZ regarding bioavailability as will be discussed later.

#### 3.7.3. Histopathological Findings

**Figure 5:**
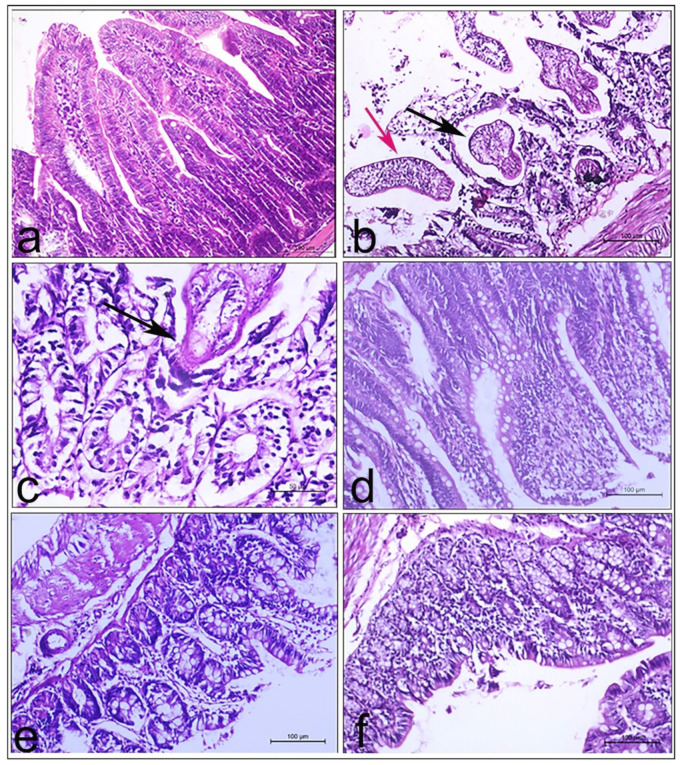
Representative intestinal sections of rats from all groups. (**a**–**f**): intestinal tissues stained with Hematoxylin and Eosin (H&E). Magnification ×100. (**a**) normal control group showed the normal intestinal villi and crypts. (**b**) intestinal section of infected non-treated control group manifested cysticercoid (black arrow) and immature worms (red arrow) surrounded by mononuclear cells infiltration. (**c**) crude PRZ (12.5 mg/kg) treated group revealed parasitic sections attached to the intestinal mucosa (arrow). (**d**–**f**) PRZ-PMMs (12.5 mg/kg), crude PRZ (25 mg/kg) and PRZ-PMMs (25 mg/kg) treated groups, respectively, presented variable degrees of submucosal parasitic granulomatous reaction associated with no parasitic sections.

The normal control group of rats showed normal histological structure of the intestinal wall composed of mucosal lining (villi and crypts), lamina propria, submucosa and musculosa, Figure 5a. Meanwhile, intestines of rats of the positive control group revealed numerous sections of tapeworms with trapezoidal proglottids being attached to a scolex armed with four suckers and hooks by means of a slender neck. Additionally, encysted worms and cysticercoid larvae were embedded within the intestinal mucosa by means of scolex having the morphological features typical for *H. nana*. A plausible explanation for the presence of cysticercoids and immature worms in the histopathological sections of these rats might be due to the autoinfection. The latter infection route of *H. nana* was confirmed in many hosts [74,75]. Mononuclear cell infiltrations including eosinophils were found within the lamina propria and also around the sectioned parasites. Congestion and hemorrhages of the submucosal blood vessels were marked in many cases. Similarly, Bayoumy et al. [2] studied the effects of PRZ amongst immunocompetent and immunocompromised infected albino mice with *H. nana* through histopathological examination of the intestine and liver. The intestinal histopathological results of the aforementioned study indicated that infected non-treated immunocompetent mice manifested degenerated, desquamated mucosa with coexisted exaggerated mucin in the intestinal lamina besides accumulation of inflammatory cells. Additionally, the intestinal villi were severely atrophied and stunted. Fusion of villi, severe necrosis and desquamation were also present in many intestinal sections, Figure 5b. Such changes could be elucidated by toxic metabolites produced by the adult worms resulting in these alterations [76]. The current results are in harmony with those of Abdel Latif et al. [77], who claimed that *H. nana* infection caused destruction of normal villous architecture.

Rats which received 12.5 mg/kg crude PRZ showed submucosal granulomatous reaction in association with the detected parasitic sections, while the groups of rats which were treated with 25 mg/kg PRZ suspension, 12.5 and 25 mg/kg PRZ-PMMs presented no parasitic sections, although the features of inflammatory granulomatous reaction were present, but congestion and edema subsided, Figure 5d–f. This remarkable improvement might be due to the reduction and expulsion of worms.

### 3.8. In Vitro Ovicidal Activity

**Figure 6:**
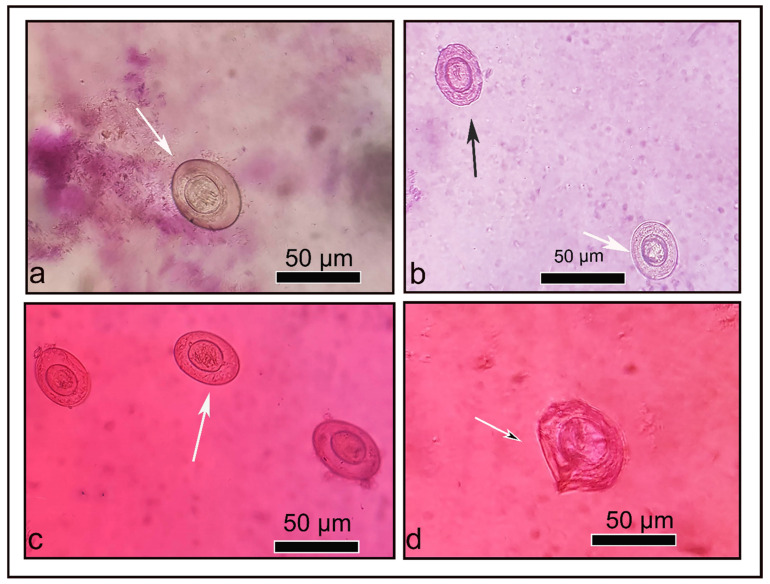
Ovicidal activity of crude PRZ and PRZ-PMMs against *H. nana* eggs. (**a**) viable non-stained untreated eggs. (**b**) crude PRZ (50 µg/mL) treated eggs, non-viable red stained eggs (black arrow) and viable non-stained eggs (white arrow). (**c**) PRZ-PMMs (25 µg/mL) treated eggs, non-viable red stained. (**d**) PRZ-PMMs (50 µg/mL) treated eggs, non-viable red stained and destructed shell.

**Figure 7:**
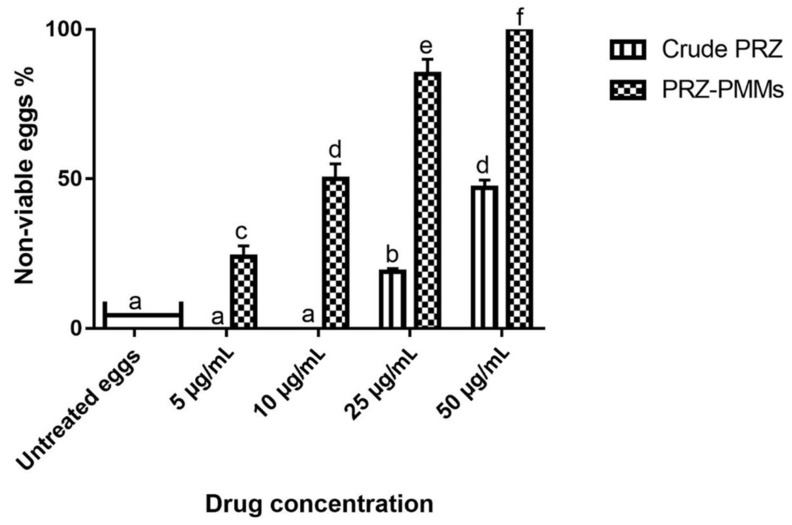
Non-viable eggs percentages in crude PRZ- and PRZ-PMMs-treated groups at different concentrations. Different letters on bars revealed a statistical difference at *p* < 0.05.

The in vitro efficacy of PRZ and its nanoformulation against *H. nana* eggs was assessed using Fuchsin vital stain [40]. Chausov’s method [40] was utilized to test the viability of the eggs. Mature eggs were translucent with refractile hooks and a moving embryo (movement is quickly lost) as well as various colors were detected. Eggs that were viable resisted the stain and appeared yellowish against red background. Dead eggs, on the other hand, absorbed the red stain and took on a completely red appearance, along with deformed hooks or outlines inside. Additionally, some eggs had only red-stained shells but still contained a healthy yellow embryo. These eggs might have already begun to be affected by the treatment, with their outer shells losing the ability to block the red stain, but their inner shells and embryo had remained intact. PRZ and PRZ-PMMs were used at concentrations of 0, 5, 10, 25 and 50 µg/mL. The viability and morphological changes of the eggs were determined. The untreated eggs were viable, had clear hexacanth embryos, non-damaged shells, and were yellow to brown in color. Meanwhile, in crude PRZ-treated eggs, non-viable eggs percentages were 19 and 47% at 25 and 50 µg/mL, respectively. The non-viable eggs were red-stained and manifested mild shell deformities and embryo damage. However, in PRZ-PMMs-treated eggs, non-viable eggs were 24, 50, 85 and 100% at 5, 10, 25 and 50 µg/mL, respectively. Moreover, shell destruction and loss of embryo were clear at 50 µg/mL, Figure 6 and Figure 7.

Overall, our results disclosed that PRZ-PMMs exhibited higher ovicidal activity than crude PRZ against *H. nana* eggs. Also, the direct effect of PRZ-PMMs formulation on *H. nana* eggs is considered a significant indication of drug efficacy where its activity is dose dependent. As has already been outlined, PRZ-PMMs at 25 and 50 µg/mL increased the percent of dead eggs to 85 and 100% versus 19 and 47% for conventional PRZ, respectively. Such findings highlight the direct effect of PRZ-PMMs on the egg shell in addition to the consolidated permeability of the employed nano-cargo.

### 3.9. Pharmacokinetic Investigations

**Figure 8:**
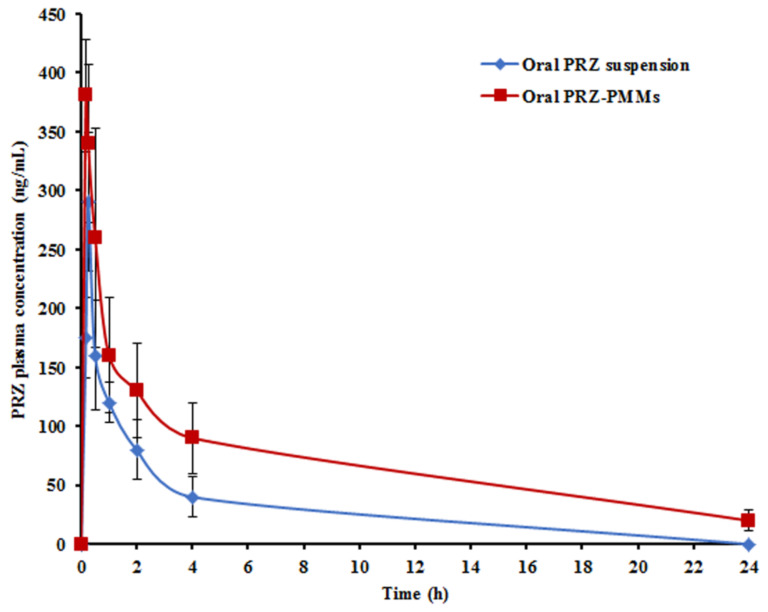
Mean PRZ plasma concentrations in rats after oral administration of the optimum micellar formulation and drug suspension (*n* = 6).

For comparing the pharmacokinetic profiles of PRZ-PMMs and PRZ suspension as a control, pharmacokinetic studies of both PRZ formulations following a single oral dose of 12.5 mg/kg utilizing male Wistar albino rats were executed adopting LC-MS/MS method. The time course of PRZ concentration in plasma is illustrated in Figure 8, and the average pharmacokinetic parameters, as estimated by non-compartmental analysis, are listed in Table 10 for both formulations. The C_max_ and AUC_0–∞_ of PRZ in rat plasma were 290.34 ± 28.53 ng/mL and 565.96 ± 102.18 ng h/mL as well as 380.75 ± 47.67 ng/mL and 1939.53 ± 289.73 ng h/mL after oral administrations of PRZ suspension and PRZ-PMMs, respectively. The oral micellar administration displayed a mild elevation in the C_max_ (approximately 1.31-fold), whereas a remarkable rise was recorded for the AUC_0–∞_ (nearly 3.43-fold) when compared with the correspondent average values of the oral suspension, *p* < 0.05. The T_max_ of PRZ suspension was 0.24 ± 0.03 h, which was significantly dwindled to 0.18 ± 0.03 h for PRZ-PMMs, demonstrating that PRZ-PMMs would be quickly absorbed upon oral administration, *p* < 0.05. Though PRZ-PMMs disclosed an in vitro extended-release pattern, the earlier T_max_ recommended fast PRZ absorption. This discrepancy could be argued in light of dissimilar conditions that evoked micellar dissociation and drug efflux with in vivo dilution which might be hardly simulated in vitro [56]. Additionally, the more intricate GIT milieu, comprising bile pigments as well as digestive enzymes, could hasten PRZ in vivo release. Moreover, sudden potential interactions of micelles with the GIT surface would prompt the absorption of PRZ. The enumerated results are in a close agreement with those by Wang et al. [78] who informed shorter T_max_ value following oral administration of curcumin-laden PMMs versus curcumin solution. 

As indicated in Table 10, PRZ-PMMs was associated with significantly longer (*p* < 0.05) mean residence time (MRT), signaling a 3.65-fold greater value than that of PRZ suspension. The average PRZ T_1/2_ estimate from PMMs (9.12 ± 1.17 h) was significantly longer than that from the oral suspension (1.11 ± 0.04 h), *p* < 0.05. These findings could denote the capacity of the optimum PMMs to accomplish a sustained PRZ plasma profile following oral administration relative to the oral drug suspension. Basalious and Shamma [79] reported prolonged blood circulatory times of PMMs greater than other nano-cargos involving stealthy-vesicles enriched with equivalent number of PEO moieties. Contrasted with PRZ oral suspension, the micellar nano-cargo recorded an F_rel_ of 342.70%.

Summing up, the above-mentioned findings manifested superior PRZ bioavailability following oral administration of PMMs, as they could collectively confer diverse privileges: (i) the hydrophobic micellar core would function as a solubilization reservoir for hydrophobic drugs such as PRZ [46]; (ii) the stealthy corona (PEO block of LF127/PEG chains of GL44) could circumvent opsonin adsorption as well as recognition by the reticuloendothelial system and, thus, donate a longer in vivo residence time [80,81]; (iii) the strikingly miniaturized size of PMMs might contribute to extended plasma circulation time by escaping the hepatic phagocytic system scavenging and bypassing the inter-endothelial splenic filtration [56,82,83]; (iv) inhibition of both cytochrome P450 metabolism and P-glycoprotein efflux which warranted higher drug plasma levels [52,84]; (v) the potential lymphatic transportation pathway of PRZ-PMMs [78]; and (vi) great payload capacity, feeble immunogenicity and diminished cytotoxicity [81]. Although LF127/GL44 polymers are non-biodegradable, substances with molecular weight < 15 kDa are often filtered through the kidney and excreted in urine [53]. Largely, the findings of the pharmacokinetic analysis assured the commendable aptitude of PMMs to snowball PRZ oral bioavailability.

## 4. Conclusions

In this study, newly-explored PMMs constituted of LF127/GL44 were assembled entrapping the hydrophobic broad-spectrum anthelminthic drug, PRZ, applying Box-Behnken design. The higher drug payload and EE% donated by the tailored optimal micellar formulation confer a greater solubilization aptitude for sparsely-soluble drugs. Also, the prolonged PRZ release pattern from the optimized micellar nanosystem alongside its typically minute size (<20 nm) proposed its promising potential in controlled/targeted drug delivery. Moreover, a single 12.5 mg/kg oral dose of PRZ-PMMs significantly boosted PRZ anthelmintic activity in *H. nana*-infected rats with good amelioration of intestinal histopathological changes. Such activity was coupled with markedly reinforced bioavailability as well as extended MRT and T_1/2_ of the optimum PRZ-PMMs compared to PRZ suspension. Thus, the designated PRZ-PMMs presented a safe and efficacious oral nanovector for hymenolepiasis tackling.

## Figures and Tables

**Table 1:** Variables of Box-Behnken design for PRZ-PMMs optimization.

Factor	Level of Variables		
	Low (−1)	Medium (0)	High (+1)
Independent variables			
*X*_1_: LF127 concentration (% *w*/*v*)	1.5	2	2.5
*X*_2_: GL44 concentration (% *w*/*v*)	1.5	1.75	2
*X*_3_: PRZ concentration (mg)	10	15	20
Dependent variables	Constraints		
*Y*_1_: EE%	Maximize		
*Y*_2_: micelle size (nm)	Minimize		
*Y*_3_: Q_24_ (%)	Maximize		

PRZ: praziquantel; PMMs: polymeric mixed micelles; LF127: Lutrol F127; GL44: Gelucire 44/14; EE%: entrapment efficiency percent; Q_24_: accumulative % release over 24 h.

**Table 2:** PRZ-PMMs experimental runs, causal variables and observed responses according to Box-Behnken statistical design.

Formulation	Independent Variables			Dependent Variables		
	*X*_1_ (% *w*/*v*)	*X*_2_ (% *w*/*w*)	*X*_3_ (mg)	*Y*_1_ (%)	*Y*_2_ (nm)	*Y*_3_ (%)
F1	1.5	1.5	15	88.93 ± 3.22	28.70 ± 3.76	55.62 ± 5.83
F2	1.5	1.75	20	94.58 ± 4.05	27.21 ± 3.22	65.05 ± 7.86
F3	1.5	2	15	81.03 ± 5.31	33.13 ± 5.91	50.21 ± 5.72
F4	2.5	2	15	64.27 ± 2.67	59.17 ± 9.82	35.67 ± 1.62
F5 *	2	1.75	15	85.71 ± 3.93	13.54 ± 2.33	74.16 ± 6.22
F6	2.5	1.75	20	78.57 ± 4.23	41.36 ± 8.23	40.19 ± 2.64
F7 *	2	1.75	15	84.53 ± 5.17	12.98 ± 2.51	75.67 ± 4.79
F8 *	2	1.75	15	87.39 ± 3.53	15.11 ± 2.68	72.88 ± 5.43
F9	2.5	1.5	15	71.90 ± 1.76	45.98 ± 7.93	38.13 ± 2.47
F10	2	2	20	76.01 ± 5.56	27.51 ± 5.46	50.84 ± 4.06
F11	1.5	1.75	10	90.02 ± 4.78	25.09 ± 3.81	69.86 ± 5.36
F12	2	2	10	73.25 ± 5.16	24.78 ± 4.79	55.65 ± 4.29
F13	2.5	1.75	10	75.41 ± 3.36	37.80 ± 3.39	43.31 ± 3.45
F14	2	1.5	20	84.39 ± 2.45	22.87 ± 4.50	62.98 ± 4.28
F15	2	1.5	10	82.19 ± 4.60	19.67 ± 4.65	67.55 ± 6.65

PRZ: praziquantel; PMMs: polymeric mixed micelles; *X*_1_: LF127 concentration (% *w*/*v*); *X*_2_: GL44 concentration (% *w*/*v*); *X*_3_: PRZ concentration (mg); *Y*_1_: entrapment efficiency percent; *Y*_2_: micelle size (nm); *Y*_3_: accumulative release over 24 h (%). Data represent mean ± SD (*n* = 3). * Indicates the center point of the design.

**Table 3:** Regression analysis results for PRZ-PMMs responses *Y*_1_, *Y*_2_ and *Y*_3_ for data fitting to various models.

Model	Adequate Precision	R^2^	Adjusted R^2^	Predicted R^2^	SD	% CV	*p* Value	Remarks
Response (*Y*_1_)								
Linear	17.42	0.7510	0.7318	0.6731	4.16	5.12	<0.0001	-
2FI	13.69	0.7516	0.7102	0.6192	4.33	5.32	<0.0001	-
Quadratic	77.91	0.9922	0.9900	0.9870	0.80	0.99	<0.0001	Suggested
Response (*Y*_2_)								
Linear	6.57	0.3160	0.2634	0.1240	11.00	37.92	0.0018	-
2FI	5.19	0.3246	0.2121	0.0167	11.37	39.22	0.0213	-
Quadratic	64.42	0.9917	0.9894	0.9865	0.05	1.39	<0.0001	Suggested
Response (*Y*_3_)								
Linear	7.60	0.3923	0.3456	0.2373	11.12	19.44	0.0002	-
2FI	5.97	0.3934	0.2923	0.1517	11.56	20.22	0.0043	-
Quadratic	86.16	0.9959	0.9948	0.9921	2.77	4.84	<0.0001	Suggested

PRZ: praziquantel; PMMs: polymeric mixed micelles; *Y*_1_: entrapment efficiency percent; *Y*_2_: micelle size (nm); *Y*_3_: accumulative release over 24 h (%); R^2^: coefficient of determination; SD: standard deviation; CV: coefficient of variation.

**Table 4:** The characteristic physicochemical properties of the investigated PRZ-PMMs formulations.

Formulation	DL%	PD%	PDI
F1	4.23 ± 0.29	0.25 ± 0.02	0.18
F2	5.48 ± 0.32	0.24 ± 0.03	0.26
F3	3.33 ± 0.27	0.32 ± 0.04	0.32
F4	2.07 ± 0.11	0.66 ± 0.03	0.13
F5 *	3.30 ± 0.20	0.11 ± 0.02	0.40
F6	3.53 ± 0.32	0.47 ± 0.03	0.37
F7 *	3.25 ± 0.14	0.10 ± 0.02	0.12
F8 *	3.36 ± 0.23	0.12 ± 0.01	0.22
F9	2.60 ± 0.25	0.62 ± 0.05	0.16
F10	3.62 ± 0.31	0.18 ± 0.02	0.20
F11	2.69 ± 0.13	0.20 ± 0.04	0.38
F12	1.79 ± 0.24	0.16 ± 0.01	0.16
F13	1.73 ± 0.16	0.41 ± 0.02	0.39
F14	4.56 ± 0.36	0.15 ± 0.05	0.27
F15	2.28 ± 0.12	0.13 ± 0.03	0.45

PRZ: praziquantel; PMMs: polymeric mixed micelles; DL%: drug-loading percent; PD%: precipitated drug percent; PDI: polydispersity index. Listed data are mean values ± SD (*n* = 3). * Indicates the center point of the design.

**Table 5:** Composition, observed and prognosticated responses for the optimized PRZ-PMMs formulation.

Factor	Optimal Value	Response Variable	Observed Value	Prognosticated Value	% Prediction Error ^a^
*X*_1_	1.79	*Y*_1_	86.29	89.78	−4.04
*X*_2_	1.68	*Y*_2_	15.18	14.29	5.86
*X*_3_	15.85	*Y*_3_	78.22	81.32	−3.96

PRZ: praziquantel; PMMs: polymeric mixed micelles; *X*_1_: LF concentration (% *w*/*v*); *X*_2_: GL44 concentration (% *w*/*v*); *X*_3_: PRZ concentration (mg); *Y*_1_: entrapment efficiency percent; *Y*_2_: micelle size (nm); *Y*_3_: accumulative release over 24 h (%). ^a^ Calculated as [Observed − Prognosticated/Observed] × 100.

**Table 6:** Storage stability of PRZ-PMMs.

Time (Months)	EE (%)	Micelle Size (nm)	ζ Potential (mV)	Q_24_ (%)
0	86.29 ± 3.26	15.18 ± 2.93	−10.35 ± 1.68	78.22 ± 4.01
1	84.97 ± 2.65	16.21 ± 3.01	−9.57 ± 1.22	76.37 ± 3.23
2	84.02 ± 1.44	17.32 ± 2.97	−8.91 ± 1.80	75.11 ± 2.59
3	83.57 ± 3.37	18.74 ± 4.10	−8.08 ± 1.13	73.81 ± 2.86

PRZ: praziquantel; PMMs: polymeric mixed micelles; EE: entrapment efficiency; Q_24_: accumulative % release over 24 h. Data are mean values (*n* = 3) ± SD, *p* ˂ 0.05 for one way ANOVA followed by Dunnett’s multiple comparison test.

**Table 7:** Mean egg count and percentages of reduction among the different groups.

Experimental Group	Mean Egg Count			Percentage Reduction of Egg Count
	Pretreatment	1st Day after Treatment	3rd Day after Treatment	
Infected non-treated control (group I)	10,900.00 ± 70.71	10,966.60 ± 248.33	10,886.60 ± 473.57	0%
Crude PRZ 25 mg/kg (group II)	10,884.00 ± 138.85	0.00 ± 0.00 ^a,c^	0.00 ± 0.00 ^a,c^	100%
Crude PRZ 12.5 mg/kg (group III)	10,832.00 ± 204.13	1033.20 ± 40.83 ^a,b,d,e^	966.60 ± 40.83 ^a,b,d,e^	90.6–91.1%
PRZ-PMMs 25 mg/kg (group IV)	10,852.00 ± 123.57	0.00 ± 0.00 ^a,c^	0.00 ± 0.00 ^a,c^	100%
PRZ-PMMs 12.5 mg/kg (group V)	10,942.00 ± 295.50	0.00 ± 0.00 ^a,c^	0.00 ± 0.00 ^a,c^	100%

PRZ: praziquantel; PMMs: polymeric mixed micelles. Values are mean ± SD, with the number of rats = 10 for each group. *p* < 0.05 was considered significant according to the post-hoc test: ^a^, versus group I; ^b^, versus group II; ^c^, versus group III; ^d^, versus group IV; ^e^, versus group V.

**Table 8:** Worm burden and its percentage reduction among the different groups.

Parameter	Infected Non-Treated Control (Group I)	Crude PRZ 25 mg/kg (Group II)	Crude PRZ 12.5 mg/kg (Group III)	PRZ-PMMs 25 mg/kg (Group IV)	PRZ-PMMs 12.5 mg/kg (Group V)
Worm count	62.20 ± 2.17	0.00 ± 0.00 ^a^	16.60 ± 1.14 ^a,b,d,e^	0.00 ± 0.00 ^a,c^	0.00 ± 0.00 ^a,c^
Percentage reduction of worm count	0%	100%	73.3%	100%	100%

PRZ: praziquantel; PMMs: polymeric mixed micelles. Worm numbers per rat were expressed as mean ± SD (*n* = 10). *p* < 0.05 was considered significant according to the post-hoc test: ^a^, significant difference versus group I; ^b^, versus group II; ^c^, versus group III; ^d^, versus group IV; ^e^, versus group V.

**Table 9:** The effect of crude PRZ and PRZ-PMMs on liver and kidney functions in different studied groups.

Group	ALT (IU/L)	AST (IU/L)	Creatinine (mg/dL)	Urea (mg/dL)
Normal control (group VI)	22.33 ± 9.29	100.67 ± 15.14	0.59 ± 0.02	28.00 ± 2.00
Crude PRZ 25 mg/kg (group II)	40.67 ± 4.73	126.00 ± 4.36	0.91 ± 0.16 ^a^^,d,e^	42.00 ± 2.65 ^a^^,c,d,e^
Crude PRZ 12.5 mg/kg (group III)	35.67 ± 10.07	104.67 ± 13.61	0.73 ± 0.09	33.00 ± 2.65 ^b^
PRZ-PMMs 25 mg/kg (group IV)	31.33 ± 7.09	113.33 ± 20.31	0.66 ± 0.04 ^b^	29.33 ± 5.03 ^b^
PRZ-PMMs 12.5 mg/kg (group V)	21.00 ± 3.61	111.67 ± 18.93	0.57 ± 0.06 ^b^	31.00 ± 2.65 ^b^

PRZ: praziquantel; PMMs: polymeric mixed micelles. Data expressed as mean ± SD (*n* = 10). *p* < 0.05 was considered significant according to the post-hoc test: ^a^, versus group VI; ^b^, versus group II; ^c^, versus group III; ^d^, versus group IV; ^e^, versus group V.

**Table 10:** Mean pharmacokinetic parameters in rat plasma after a single oral dose (12.5 mg/kg) of PRZ suspension or PRZ-PMMs.

Pharmacokinetic Parameter	Mean ± SD	
	PRZ Suspension	PRZ-PMMs
C_max_ (ng/mL)	290.34 ± 28.53	380.75 ± 47.67 ^a^
T_max_ (h)	0.24 ± 0.03	0.18 ± 0.03 ^a^
K_elim_ (h^−1^)	0.6243 ± 0.0412	0.0760 ± 0.0062 ^a^
T_1/2_ (h)	1.11 ± 0.04	9.12 ± 1.17 ^a^
AUC_0–t_ (ng h/mL)	389.73 ± 95.27	1676.10 ± 367.34 ^a^
AUC_0–∞_ (ng h/mL)	565.96 ± 102.18	1939.53 ± 289.73 ^a^
MRT (h)	3.05 ± 0.46	11.12 ± 3.02 ^a^
F_rel_ (%)	--	342.70

PRZ: praziquantel; PMMs: polymeric mixed micelles. Each value is the mean ± SD of six separate determinations. Using two-sided Student’s *t*-test assuming equal variance. ^a^ *p* < 0.05 versus oral PRZ suspension.

## Data Availability

All processed data in this work are incorporated into the article.

## Supplementary Materials

The following supporting information can be downloaded at: https://www.mdpi.com/article/10.3390/pharmaceutics14102023/s1, Figure S1: In vitro release profiles of PRZ from crude drug and various PMMs formulations: (a) F1-F8 and (b) F9-F15; Table S1: Kinetic parameters of PRZ release from the micellar dispersions.
