# Supplementary material for: Tunable Polymeric Mixed Micellar Nanoassemblies of Lutrol F127/Gelucire 44/14 for Oral Delivery of Praziquantel: A Promising Nanovector against Hymenolepis nana in Experimentally-Infected Rats
Source: Pharmaceutics. 2022 Sep 23;14(10):2023. doi: 10.3390/pharmaceutics14102023 (PMC9608995; doi:10.3390/pharmaceutics14102023)
Supplement: Supplementary file 1 [file pharmaceutics-14-02023-s001.zip › pharmaceutics-1899217-supplementary.pdf]

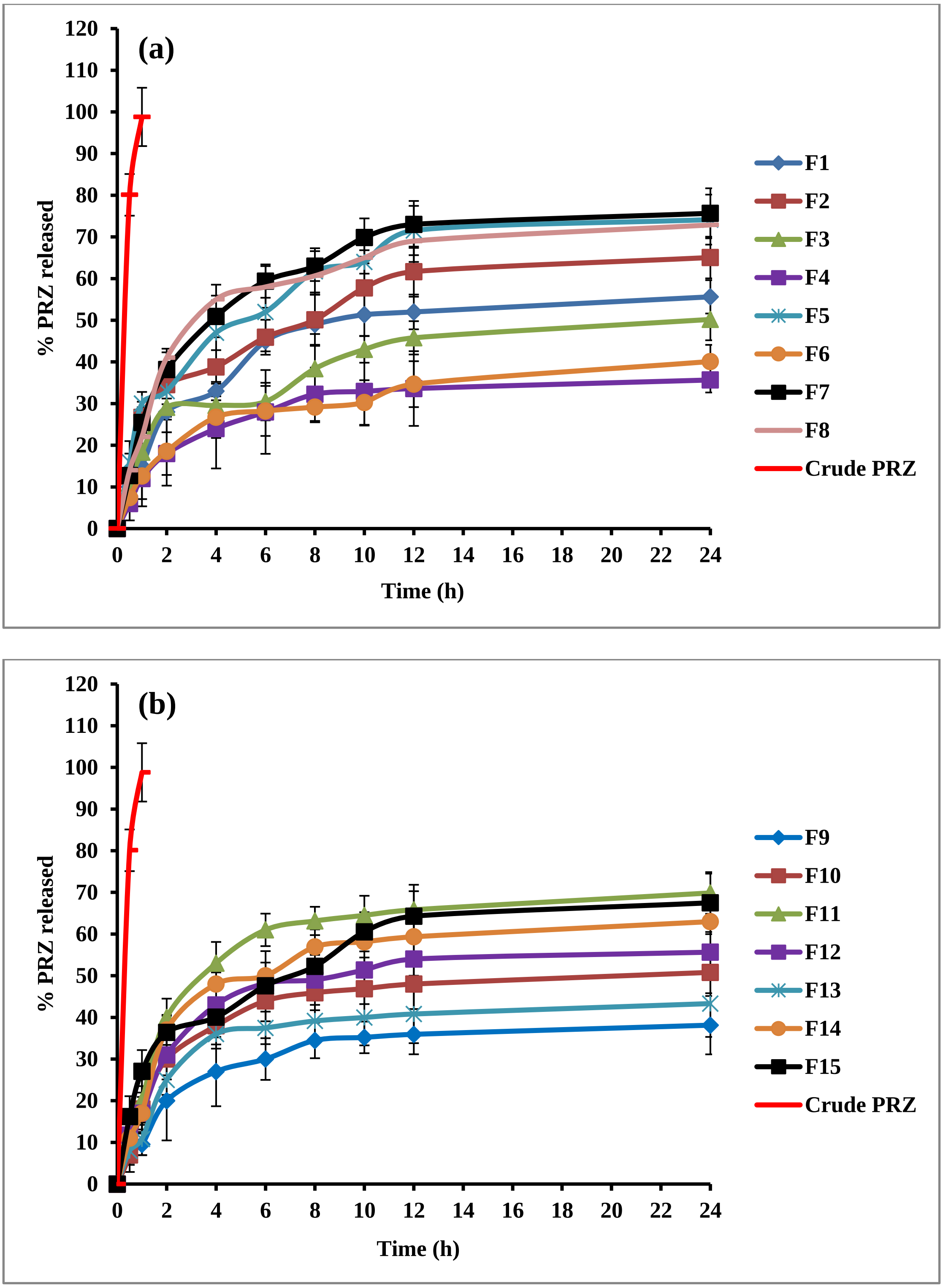

Figure S1. In vitro release profiles of PRZ from crude drug and various PMMs formulations: (a) F1-F8 and (b) F9-F15.

Table S1. Kinetic parameters of PRZ release from the micellar dispersions

Formulation	Zero-order kinetics $Q = K_o\mathrm{t}$	First-order kinetics $LnQ = K_1 t$	Higuchi kinetics $Q = K\mathrm{t}^{1/2}$	Release order
		R^2		
F1	0.8297	0.8129	0.8788	Higuchi kinetics
F2	0.8731	0.8630	0.9501	Higuchi kinetics
F3	0.8429	0.8441	0.8987	Higuchi kinetics
F4	0.8608	0.9288	0.9759	Higuchi kinetics
F5	0.8439	0.9411	0.9169	First-order
F6	0.8762	0.9325	0.9763	Higuchi kinetics
F7	0.8565	0.8745	0.8062	First-order
F8	0.8828	0.9679	0.8878	First-order
F9	0.8865	0.9455	0.9853	Higuchi kinetics
F10	0.9338	0.9022	0.8964	Zero order
F11	0.7895	0.8024	0.8730	Higuchi kinetics
F12	0.9532	0.9343	0.9765	Higuchi kinetics
F13	0.9154	0.9077	0.8268	Zero-order
F14	0.9363	0.9557	0.9874	Higuchi kinetics
F15	0.9541	0.9566	0.9669	Higuchi kinetics

Data represent mean ± SD (n = 3).
